# Xanthophylls lutein and zeaxanthin modify gene expression and induce synthesis of hyaluronan in keratinocyte model of human skin

**DOI:** 10.1016/j.bbrep.2015.08.012

**Published:** 2015-08-21

**Authors:** Rasia Li, Stephen D. Turner, David L. Brautigan

**Affiliations:** a Center for Cell Signaling, Department of Microbiology, Immunology & Cancer Biology, University of Virginia School of Medicine, Charlottesville, VA 22908, United States; b Bioinformatics Core, University of Virginia School of Medicine, Charlottesville, VA 22908, United States

**Keywords:** Hyaluronic acid, Glycosaminoglycan, Blyscan, Serpin, EpiDerm

## Abstract

**Background:**

Clinical trials report benefits of the xanthophylls lutein and zeaxanthin for skin health. Here a keratinocyte culture was used to investigate the effects of *in vitro* xanthophyll treatment on gene expression and biochemical pathways.

**Methods:**

We employed the EpiDerm tissue model, Affymetrix Human Genome Array U113, bioinformatics analyses, qPCR validation and biochemical assays for glycosaminoglycans.

**Results:**

We discovered 176 genes were significantly (*p*<0.05) down-regulated (log 2FC>2) and 47 genes were significantly up-regulated. Among the down-regulated genes we validated by qPCR marked reduction in expression of peptidase inhibitors. Bioinformatic analysis of the up-regulated genes implicated biosynthetic pathways for glycosaminoglycans. We assayed but found no increase in production of sulfated glycosaminoglycans, however there was a significant increase in biosynthesis of hyaluronic acid, a non-sulfated glycan.

**Conclusions:**

The pattern of xanthophyll-regulated genes and the resulting biochemical responses can be linked with the responses observed in clinic trials.

**General significance:**

Skin health benefits from xanthophyll supplementation and this study reveals molecular mechanisms for some of the effects.

## Highlights

• Lutein and zeaxanthin treatment of Epiderm model of human skin changes global gene expression.
• More genes were down-regulated than up-regulated
• Up regulated genes were linked to glycosylaminoglycan synthesis and more hyaluronan was produced
• These changes correspond to improved skin quality seen in clinical trials.

## Graphical abstract

Clustering and display of relative identities based on differences in gene expression patterns between three control and three xanthophyll treated samples of EpiDerm human skin.

**Figure:**
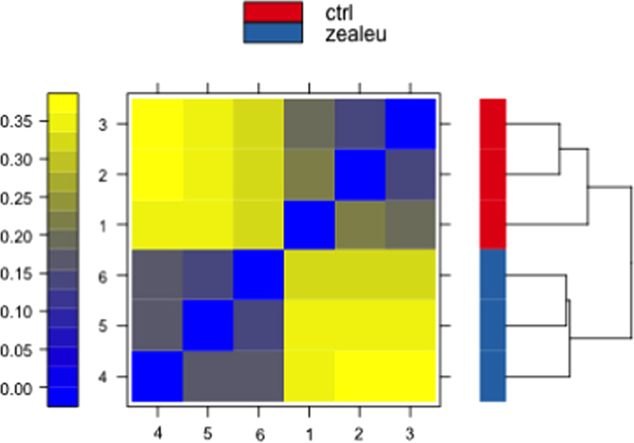

## 1 Introduction

Lutein and zeaxanthin are xanthophylls, a class of isomeric tetraterpenoid carotenoids characterized by their yellow color and hydroxyl group substituents. Like other carotenoids, xanthophylls serve as pigments in photosynthetic organisms and are not synthesized by animals and therefore must be obtained from the diet, typically from leafy vegetables such as spinach and kale, or egg yolks where they are concentrated from feed sources [1], [2], [3], [4].

Lutein and zeaxanthin become concentrated in the *macea lutea*, the focal center of the eye, and are measured as macular pigment optical density. Lutein and zeaxanthin absorb blue light to reduce oxidative stress in the retina and are believed to function as antioxidants that protect photoreceptor cells against free radicals produced by light and high oxygen tension [5]. A major clinical trial (AREDS2) sponsored by the US National Institutes of Health showed consumption of supplemental lutein and zeaxanthin reduced the risk of age-related macular degeneration and improved visual acuity, particularly contrast acuity [6]. Largely driven by the results of AREDS2, lutein and zeaxanthin are currently marketed as nutritional supplements for their role in macular health.

The antioxidative properties of xanthophylls also are believed to play a role in protecting the skin against light-induced damage. Lutein and zeaxanthin are found in the skin as a result of dietary intake. The ingestion and deposition of dietary antioxidants is one form of protection against radiation, as other protective measures, such as melanin protection, are triggered only after light-induced damage occurs in the skin [7], [8]. The visible wavelengths absorbed by lutein and zeaxanthin are relatively high energy, and although not in the ultraviolet range, are capable of producing free radicals in the skin [8]. Xanthophylls are thought to protect against photodamage in the skin in the same manner as they are thought to function in the retina [9]. Ingested lutein and zeaxanthin have thus far been shown to protect against UV-induced skin damage, including edema and hyperplasia, in animals [10].

A 2002 clinical trial conducted by Palombo et al. [11] demonstrated the efficacy of lutein and zeaxanthin in improving skin health. Physiological properties of skin were measured in women given oral and/or topical treatment of a lutein/zeaxanthin combination: (1) surface lipid production, (2) lipid peroxidation, (3) photoprotective activity, (4) skin elasticity, and (5) skin hydration. Both oral and topical supplementation had a positive effect on all these variables measured individually, with the largest effects found in response to the combination of oral plus topical treatments. Oral and topical administration of xanthophylls individually and combined also moderately increased photoprotective activity within the first two weeks of treatment. Topical treatment proved most effective in increasing skin elasticity. Palombo et al. attributed the increased photoprotective activity by lutein and zeaxanthin treatment to the protection they provide against free radicals. They also attributed the decrease in lipid peroxidation to the antioxidative effects of these xanthophylls [11].

We wanted to test the hypothesis that the beneficial effects on skin observed clinically were not simply due to the antioxidant and light absorbing properties of the xanthophylls. Xanthophylls chemically resemble retinoids that act though nuclear hormone receptors. Unlike retinoids, xanthophylls are not converted into vitamin A, nonetheless we suspected that they could alter patterns of gene expression. In this study, we analyzed gene expression profiles using EpiDerm an *in vitro* model of human skin and also tested for some biochemical changes that corresponded to the genes most affected by lutein and zeaxanthin.

## 2 Materials and methods

### 2.1 Cell culture and reagents

EpiDerm (EPI-212) tissue models of human keratinocytes were purchased from MatTek Corporation with provided DMEM medium. EpiDerm samples were cultured in the medium supplemented with final concentrations of 5 μM lutein and 1 μM zeaxanthin or the corresponding volume of DMSO at 37 °C for 24 h. We chose these conditions because xanthophylls have been detected at micromolar concentrations in human plasma and blood [6], [12] and the 5:1 ratio has been used as a dietary supplement in clinical studies [6] and adopted as the commercial supplement formulation. Purified lutein and zeaxanthin dissolved in DMSO were provided by Kemin Industries, Des Moines, IA.

### 2.2 Affymetrix microarray gene chip analysis

Total RNA was extracted from EpiDerm samples using RNeasy Mini Kit (QIAGEN) and submitted to the UVA Biomolecular Research Facility for Affymetrix gene chip analysis using the Human Genome Array U113. Data was analyzed by the UVA Bioinformatics Core. All data processing and analysis was done using R and Bioconductor packages. Affymetrix CEL files were imported using the *affy* package. Expression intensities were summarized, normalized, and transformed using Robust Multiarray Average algorithm [13]. Probesets were annotated using the '*GEOquery*' package. For examining differential gene expression, we fit a linear model with empirical-Bayes moderated standard errors using the *limma* package in R.

### 2.3 Pathway analysis

We employed three bioinformatic tools in the analysis of the Affymetrix array data. The first of these was Gene Set Enrichment Analysis (GSEA) that uses all genes and their fold change obtained from the Affymetrix analysis and computes an enrichment group for gene groupings [14]. A second tool was the DAVID functional annotation tool, developed by the Laboratory of Immunopathogenesis and Bioinformatics for the National Institute of Allergy and Infectious Diseases. It conducts a search using a user-inputed list of genes to identify known pathways containing those genes [15], [16]. The third tool was the ConsensusPathDB (CPDB) from the Max Planck Institute for Molecular Genetics, which includes a gene set over-representation analysis tool. It takes a user-defined gene list and searches among over-representation sets. A *p*-value and *q*-value are calculated for each set, and the involved genes are represented [17], [18]. The oPOSSUM algorithm was used to perform single-site transcription factor analysis, which produced a *Z* score, which is the number of standard deviations above or below the expected rate of occurrence of a transcription factor binding site [19], [20], [21].

### 2.4 RT-PCR validation

RNA was extracted from one biological replicate of Epiderm samples, control and xanthophyll treated, using the RNeasy Mini Kit. The purified RNA was used to synthesize cDNA, which was analyzed using the Human Drug Metabolism RT^2^ Profiler PCR array from Qiagen, which contained 16 of the downregulated genes, based on the Affymetrix expression data. We analyzed the cDNA and compared fold change (FC) in the relevant genes. Another independent RT-PCR assay was performed to verify the results of the gene expression analysis. This consisted of a custom set of primers for the top ten (most upregulated) and bottom ten (most downregulated) genes from the Affymetrix data, based on log ratios. This assay was done in duplicate for the control and xanthophyll treated samples.

### 2.5 Blyscan dye-binding assay

Sulfated glycosaminoglycan (GAG) production was measured using the Blyscan assay developed by Barbosa et al. [22]. Triplicate samples of EpiDerm tissue were treated with lutein/zeaxanthin combination for 0, 1, 2, or 3 days. Each individual tissue sample was removed from its filter insert and digested using papain following the manufacturer's instructions to release GAGs from the tissue. The Blyscan dye-binding assay was performed on both the digested tissue and culture media of each sample to determine the total GAG content. The Blyscan assay uses specific binding of 1,9-dimethylmethylene blue to sulfated GAGs and isolation of the GAG–dye complex as a pellet, followed by dissociation and quantification using spectrophotometry. Absorbance was measured with a 650 nm filter, near the maximum absorbance of the dye at 656 nm.

### 2.6 ^35^S-sulfate labeling assay

Triplicate EpiDerm samples were incubated with lutein/zeaxanthin combination for 0, 1, or 3 days, in medium with 0.7 mCi of ^35^S-sulfate. The tissue samples in the filter inserts were washed three times with PBS and the washes monitored for removal of unincorporated radioactivity. The tissues were removed from the inserts and digested with papain, then entire sample suspended in Scintisafe Econo 1 Cocktail and the ^35^S content determined using a Beckman LS-6500 liquid scintillation counter.

### 2.7 Hyaluranon assay

Quantification of hyaluranon (HA) was performed using an enzyme-linked competitive binding assay from R&D Systems. Triplicate EpiDerm samples were treated with lutein/zeaxanthin combination for 24 h, the media was removed and assayed undiluted and diluted 10×. Precoated 96-well plates were used to bind HA, which was detected by absorbance at 450 nm. A calibration curve was constructed from commercial HA standard provided. The undiluted samples exceeded the linear range, so 10× diluted samples, which were in the linear range, were used to calculate the yield of HA.

## 3 Results

### 3.1 Effects of xanthophylls

#### Supplementation on gene expression in human keratinocytes

**Fig. 1:**
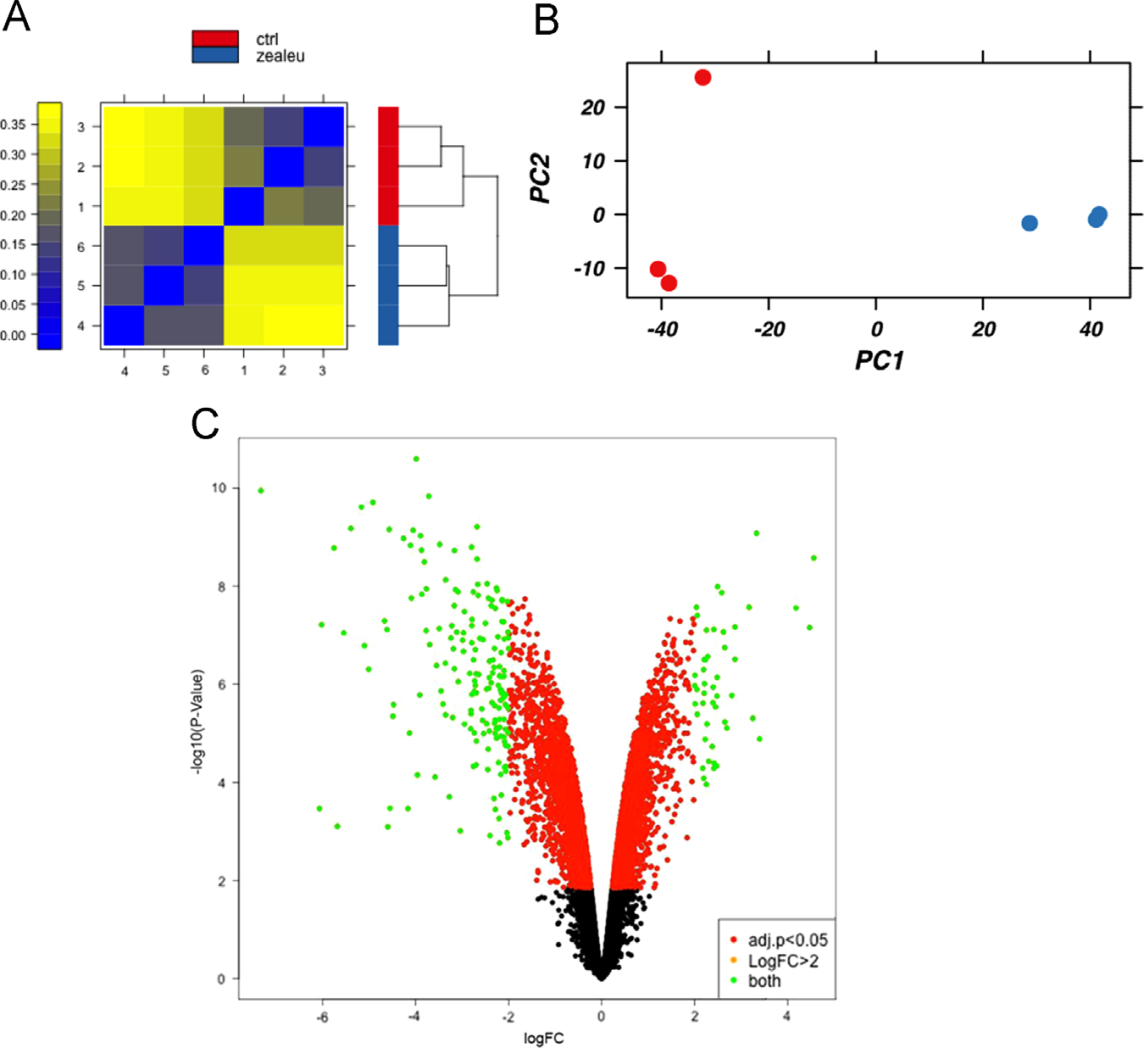
(A) Hierarchical clustering and heat map and (B) principal component analysis showing clear separation between gene expression of control and xanthophyll treated samples. Red dots represent control samples; blue dots represent xanthophyll treated samples. (C) Volcano plot maps gene expression based on fold change between control and lutein/zeaxanthin treated samples and corresponding *p*-values. Green dots represent genes identified as having statistically significantly large fold change: 47 upregulated and 176 downregulated.

The human EpiDerm keratinocyte culture system is an established *in vitro* model for human skin [23]. Using the Epi-212 multiwell plate, three wells were supplemented with lutein plus zeaxanthin and three wells were treated with DMSO as control for 24 h at 37 °C. The individual tissue specimens were extracted and RNA was isolated. Purified RNA was profiled using Agilent microcapillary electrophoresis system to ensure quality, with RIN≥9. Gene expression analysis was performed using Affymetrix Human Genome Array U133. Hierarchical clustering and principal component analysis (PCA) (Fig. 1A and B) showed clear separation between the control and experimental samples and similarities amongst the replicates.

A volcano plot was used to graphically depict the fold change (log FC on the *X* axis) and statistical significance (−log_10_(*p*-value) on the *Y* axis) of the differences in gene expression (Fig. 1C). The green dots in the upper right and left quadrants of the volcano plot show the 47 most up-regulated genes and the 176 most down-regulated genes, defined as |log(FC)|>2 and log_10_(adj. *p*-value)<0.05.

### 3.2 Pathway analysis

**Fig. 2:**
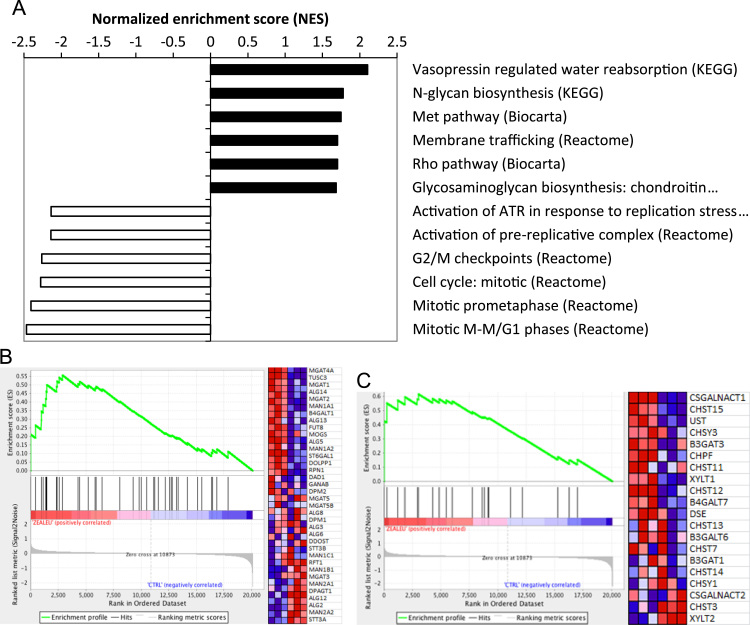
(A) Top 12 pathways identified by GSEA as upregulated or downregulated by xanthophyll treatment based on NES. Closed bars represent pathways with the highest positive NES values, and open bars represent pathways with the lowest negative NES values. Most notable are the upregulated pathways for (B) N-glycan synthesis and (C) glycosaminoglycan biosynthesis: chrondroitin sulfate. These two gene sets are shown along with a corresponding heat map in the three control and three xanthophyll sample.

Gene Set Enrichment Analysis (GSEA) uses all genes from the microarray analysis and their corresponding fold change to compute a normalized enrichment score (NES) for gene groupings based on known gene ontology sets or KEGG pathways (*14*). Of the 653 gene sets, 56 were significantly up-regulated (*p*<0.05) and two of the top sets (Fig. 2A) were related to glycan biosynthesis: N-glycan biosynthesis (NES=1.776, *p*=0.0054) and biosynthesis of chondroitin sulfate (NES=1.684, *p*=0.0096) (Fig. 2B and C). Another 68 gene sets were significantly down-regulated by xanthophyll treatment, and those with the most negative NES (Fig. 2A) included genes related to DNA replication and mitosis.

**Fig. 3:**
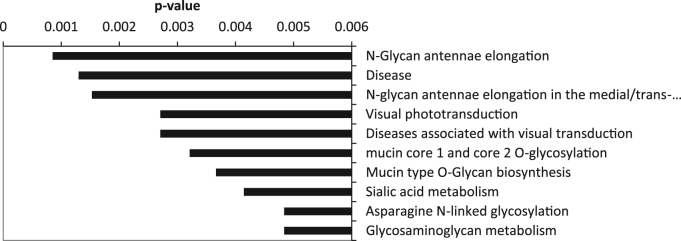
Top 10 pathways significantly upregulated by xanthophyll treatment based on ranking of *p*-values identified by CPDB.

As an alternative, we used DAVID functional annotation [15], [16] to identify pathways enriched in xanthophyll-regulated genes. The only statistically significantly up-regulated pathway was O-glycan biosynthesis. Because DAVID only identified one up-regulated pathway, we also employed ConsensusPathDB functional annotation from the Max Planck Institute [17], [18] to identify additional pathways up-regulated by xanthophyll treatment. Pathways related to N-glycan, O-glycan and glycosaminoglycan synthesis and glycosylation showed statistically significant enrichment (Fig. 3).

### 3.3 Experimental validation of gene expression changes

We carried out an independent biological replicate experiment, exposing EpiDerm to the 5:1 ratio of xanthophylls. The RNA was analyzed using qPCR for the ten genes with the highest positive fold change and 10 genes with the largest negative fold change. Only three of the 10 most up-regulated genes showed positive fold change by qPCR, notably chondroitin sulfate N-acetylgalactosaminyltransferase-1. On the other hand, eight of the 10 most down-regulated genes showed negative fold change by qPCR, including robust reduction of multiple peptidase inhibitors (FETUB, SPINK7, and SERPINA12).

The GSEA gene set (Drug metabolism: cytochrome P450) had a negative enrichment score, and this set contains multiple genes for glutathione S-transferases, cytochrome P450, and flavin monooxygenases. The P450 and flavin monooxygenase genes were presented in the Human Drug Metabolism RT^2^ Profiler PCR array that we purchased to analyze RNA from control and xanthophyll treated EpiDerm. However, we noted that many genes in this profiler array have relatively low expression levels in keratinocytes, and only 10 of the 84 genes in this array showed significant fold change in response to xanthophylls. The profiler array included 16 genes that showed significant down-regulated in the Affymetrix analysis, but none of these were validated by the profiler array.

### 3.4 Effects of xanthophylls on glycosaminoglycan production

Glycosaminoglycans (GAGs) are a class of primarily O-linked glycans known to play a role in skin hydration and wound healing. They can be divided into two classes: (1) sulfated, which consists of chondroitin sulfate, dermatan sulfate, keratan sulfate, heparin sulfate, and heparin, and (2) non-sulfated, which consists only of hyaluronan [24].

**Fig. 4:**
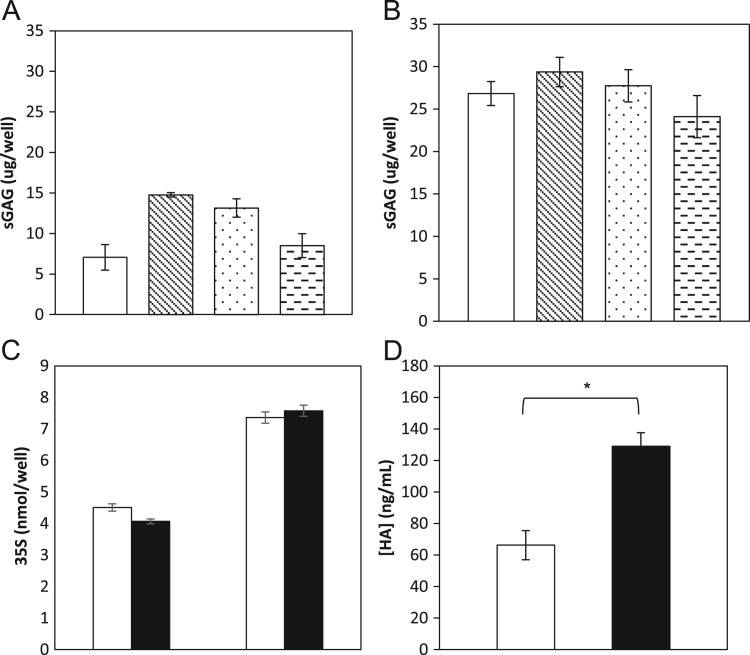
(A) sGAGs produced per EpiDerm well measured in the media by the Blyscan sulfated glycosaminoglycan assay (left to right: control – open bar, 1 day xanthophyll treatment, 2 day xanthophyll treatment, 3 day xanthophyll treatment). No significant difference between control and treated samples (*p*=0.336). (B) sGAGs bound to EpiDerm tissue released by papain digest, then measured by the Blyscan sulfated glycosaminoglycan assay (left to right: control, 1 day xanthophyll treatment, 2 day xanthophyll treatment, 3 day xanthophyll treatment). (C) sGAG produced per EpiDerm tissue digest at 24 and 72 h measured by ^35^S-sulfate labeling. No significant difference was found between control (open bars) and treated samples (filled bars) at 24 or at 72 h (*p*=0.141, *p*=0.646). (D) Hyaluronan in EpiDerm tissue media at 24 h measured by an enzyme-linked competitive binding assay (control, open bar; xanthophyll treated, filled bar). A significant increase in hyaluronan production was found as a result of lutein/zeaxanthin treatment (*n*=3, *p*=0.0019).

Sulfated glycosaminoglycans (sGAGs) produced by the EpiDerm cultures were quantified using the Blyscan dye-binding assay and by radioactive ^35^S-sulfate metabolic labeling. For the Blyscan assay, a fresh set of EpiDerm samples were treated in triplicate with vehicle for three days (control) or xanthophylls for 1, 2, or 3 days. Media were assayed for sGAG content (Fig. 4A). Within 24 h, the EpiDerm produced 15 µg of sGAGs per well, which after two days was 13 µg per well and after 3 days was 8 µg per well, compared to 7 µg per well in the untreated controls. We concluded that there was an initial synthesis and release followed by some degradation of the sGAGs. Total sGAG biosynthesis by these cultures was determined by combining the amount released into the media plus the amount released by papain digestion of the tissues, a standard method used for analysis of total sGAGs (Fig. 4B). These results showed that about half of the total sGAGs were retained in the tissues (15 µg/well *vs.* 30 µg/well). Over the three days of incubation, a one-way ANOVA test showed no significant difference between control and xanthophyll-treated samples (*F*(3,8)=1.31, *p*=0.336).

We also measured sGAGs synthesis in the tissues using ^35^S-sulfate metabolic labeling. Triplicate samples of EpiDerm were treated with vehicle alone or xanthophylls for either 1 or 3 days. Tissues were removed from their inserts and washed repeatedly with PBS to remove any unincorporated ^35^S, based on monitoring radioactivity of the washes. Tissues were digested by papain to release the sGAGs, and ^35^S-content was quantified for each sample by scintillation counting. There was a greater than 50% increase in ^35^S incorporation between days 1 and 3, and student two-way *t*-tests showed no significant difference between ^35^S-incorporation into sGAGs between control and xanthophyll treated samples at either 1 or 3 days (*p*=0.141, *p*=0.646). From these experiments, we concluded that xanthophylls did not significantly increase the biosynthetic production of sGAGs.

Because we did not detect any increase in the amount of sGAGs in xanthophyll treated tissue samples compared to control samples, even though genes for these pathways were up-regulated, we assayed for hyaluronan, the non-sulfated GAG, using a enzyme-linked competitive binding assay with a standard curve generated using purified hyaluronan. Triplicate samples of EpiDerm were incubated for 1 day in control or xanthophyll supplemented media. Medium was removed and required 10× dilution for quantification of the hyaluronan. Control samples averaged a concentration of 66 ng/mL, compared to xanthophyll-treated samples that produced 130 ng/mL (Fig. 4D). A two-way student *t*-test showed a significant increase in hyaluronan production in response to xanthophyll supplementation (*p*=0.002).

The terminal step of hyaluronan synthesis is catalyzed by three isoforms of hyaluronic acid synthase (HAS1, HAS2, and HAS3) (*25*). The gene expression data from the Affymetrix array showed a significant increase in expression of HAS3 (log 2FC=0.812, adj. *p*<0.001), but not HAS1 (adj. *p*=0.630) or HAS2 (adj. *p*=0.113).

## 4 Discussion

The molecular mechanisms for the beneficial changes in the skin induced by xanthophylls (lutein+zeaxanthin) remain unknown. In this study, we used a validated *in vitro* model of human tissue, EpiDerm, to determine the effects of xanthophyll treatment on gene expression. One advantage of this model is that cells are grown in 3D on filters at a liquid–air interface, which promotes differentiation while the single cell type allows side-by-side genetic comparison for effects of treatments without the confounding presence of lymphocytes or other cell types from the dermis or subcutaneous layers of the skin. Bioinformatic analysis of the gene expression data from Affymetrix arrays revealed that there were 176 genes significantly (*p*<0.05) down-regulated (log 2FC>2) compared to 47 genes that were significantly up-regulated. The down-regulated genes are in pathways for arachidonic acid and eicosanoid metabolism and biological oxidation. In clinical trials, lipid peroxidation was observed to decrease, and photoprotective activity was observed to increase due to xanthophyll treatments in clinical trials [11]. Arachidonic acid is degraded into malondialdehyde (MDA) [32], [33], which was used as a measure of lipid peroxidation in the clinical trial. Down regulation of genes involved in arachidonic metabolism by xanthophylls would decrease MDA production, supporting the clinical observations. Arachidonic acid is also metabolized into pro-inflammatory eicosanoids, including prostaglandins and leukotrienes [34]. Reduction in expression of genes for enzymes in these pathways is consistent with the observed decrease in UV-induced erythema, as a measure of photoprotective activity by xanthophylls seen in clinical trials. We also note that the genes down-regulated by xanthophylls include a number of serpin protease inhibitor genes. Of the most down-regulated genes, a majority that were in a secondary PCR array were validated, including genes encoding extracellular peptidase inhibitors (FETUB, SPINK7, and SERPIN A12). Reduced levels of these proteins may be related to remodeling of skin [35].

Analysis of up-regulated genes pointed to enhancement of pathways for glycosaminoglycan biosynthesis. We directly assayed the biosynthetic products of these pathways. We found no evidence for xanthophyll-induced increase in production of sulfated glycosaminoglycans. On the other hand, there was a significant, almost 2-fold increase in the production of hyaluronic acid (hyaluronan), a non-sulfated glycan. We note that the amount of hyaluronan released into the medium was only a fraction of the amount of sGAGs released by the EpiDerm system. It is possible that hyaluronan remained associated with the cells. Xanthophyll induction of hyaluronan synthesis likely corresponds to the clinical observation that skin hydration and elasticity were significantly improved by xanthophyll treatments [11]. Hyaluronan is known to have high water-binding capacity and therefore plays an important role in maintaining skin hydration. There is also evidence that hyaluronan can act as a signaling molecule in response to injury to induce wound repair, and as a free radical scavenger to prevent damage to the skin [25], [26], [27], [28]. In cultured human keratinocytes expression of HAS3 was reported as low relative to HAS1, but stimulated by addition of HB-EGF [36]. A previous study by Sayo et al. [29] demonstrated that a number of non-provitamin A carotenoids, including lutein and zeaxanthin, induced hyaluronan synthesis and expression of HAS3 in human foreskin keratinocytes. Lutein and its metabolites have been proposed to act *via* retinoic acid receptors (RARs) to increase HAS3 mRNA and hyaluronan biosynthesis [29], [30], [31].

We used the oPOSSUM algorithm to perform analysis of transcription factors for the 47 genes most highly induced by xanthophylls. The statistical analysis produces a *Z* score, which is the number of standard deviations above or below the expected rate of occurrence of a transcription factor binding site [19], [20], [21]. The transcription factors PPARG:RXRA (*Z*=11.1) and RXR:RAR_DR5 (*Z*=9.90) had the highest *Z*-scores. We speculate that these two transcription factors may play a role in the up regulation of xanthophyll-induced genes. Consistent with this concept, we found relatively high expression levels for these transcription factors, PPARG (average expression=7.1) and RXRA (average expression=10.4), in the RNA extracted from EpiDerm. Whether xanthophylls act as ligands for these or other transcription factors is not known. Because many more genes are down-regulated *vs.* up-regulated by xanthophylls one has to suspect that whatever proteins are acting as receptors for xanthophylls, these ligands are promoting recruitment of co-repressors as part of their mechanism of action.

## 5 Conclusions

The xanthophylls lutein plus zeaxanthin alter gene expression in an *in vitro* model of human skin and these changes provide a mechanistic basis of the clinical benefits of xanthophylls.
